# A Community-Driven Approach to Identifying “Winnable” Policies Using the Centers for Disease Control and Prevention’s Common Community Measures for Obesity Prevention

**Published:** 2012-03-29

**Authors:** Stephanie B. Jilcott Pitts, Lauren M. Whetstone, Jean R. Wilkerson, Tosha W. Smith, Alice S. Ammerman

**Affiliations:** Department of Public Health, Brody School of Medicine, East Carolina University; East Carolina University, Greenville, North Carolina; Communities Putting Prevention to Work, Pitt County Health Department, Eat Smart Move More, Greenville, North Carolina; University of North Carolina at Chapel Hill, Chapel Hill, North Carolina; University of North Carolina at Chapel Hill, Chapel Hill, North Carolina

## Abstract

Federally funded, community-based participatory research initiatives encourage the development and implementation of obesity prevention policies. In 2009, the Centers for Disease Control and Prevention (CDC) published the Common Community Measures for Obesity Prevention (COCOMO), which include recommended strategies and measures to guide communities in identifying and evaluating environmental and policy strategies to prevent obesity. Agreeing on "winnable" policy issues can be challenging for community members. We used CDC's COCOMO to structure in-depth interviews and group discussions with local stakeholders (ie, planners, town managers, and a local community advisory council) to stimulate interest in and identify health-promoting policies for local policy and planning agendas. We first asked stakeholders to rank the COCOMO recommendations according to feasibility and likelihood of success given community culture, infrastructure, extent of leadership support, and likely funding support. Rankings were used to identify the most and least "winnable" COCOMO policy strategies. We then used questions from the evidence-based *Community Readiness Handbook* to aid discussion with stakeholders on the facilitators and barriers to enacting the most and least winnable policy options identified. Finally, we discuss potential adaptations to COCOMO for rural jurisdictions.

## Introduction

Obesity negatively affects the health of millions of Americans and substantially increases health care costs (1,2). Individual-level prevention and treatment programs have been modestly successful; environmental and policy changes are increasingly recommended to prevent obesity (3,4). Federally funded initiatives to identify and enact obesity prevention policies have increased. For example, the Communities Putting Prevention to Work (CPPW) initiative, funded by the Centers for Disease Control and Prevention (CDC), charges funded communities with "improving health behaviors by changing community environments." Without supportive policy changes, such environmental-change goals may be difficult to achieve and maintain over time, and communities need guidance in selecting the most promising, or "winnable," obesity-prevention policy strategies to fit their local context.

In 2009, CDC published 24 recommended strategies and measures, Common Community Measures for Obesity Prevention (COCOMO), to guide communities in identifying and implementing obesity prevention policy strategies (3). These recommendations are focused on the food and physical activity environments, are evidence-based, and were informed by an expert advisory committee. COCOMO recommendations are broad, ranging from increasing supermarket availability to improving access to recreational facilities (3). Each recommended strategy is accompanied by a suggested measure intended to guide public health advocates in planning and monitoring the effect of food and physical activity environmental changes (3). Although COCOMO-recommended strategies and measures are well-suited for urban areas, they may need to be adapted for use in rural areas.

COCOMO provides a comprehensive structure to guide obesity prevention policy planning and monitoring; however, to our knowledge, no systematic process for using COCOMO as a guide for policy planning among local stakeholders has been described. The purpose of this article is to present a community-driven, COCOMO-guided approach to identifying winnable local policy strategies for obesity prevention in 2 largely rural counties in eastern North Carolina. We describe use of COCOMO to structure in-depth interviews and a group discussion with local stakeholders and to learn more about how health-promoting policies could be incorporated into the local policy and planning agenda.

## The Setting: Rural Eastern North Carolina

In eastern North Carolina, Pitt and Lenoir counties are centered in the heart of the "stroke belt" that runs through the southeastern United States. Both counties have a higher percentage of medically underserved residents living below the federal poverty level than the rest of the state (Table). Greenville is a small urban center, the Pitt County seat, with an estimated population of 84,986; Kinston is the county seat of Lenoir, with an estimated population of 22,056 (9). In Pitt County, there are 3 small towns (population range: 4,615-8,586) and 6 very small towns (population range: 112-2,240); in Lenoir County, small towns range from 527 to 2,737 residents (9). Although Lenoir and Pitt counties are adjacent, Lenoir County had a more than 5% net decrease in population between 2000 and 2009, and Pitt County had an 18% net increase in population during that period (9).

We used COCOMO for 2 initiatives. Pitt County was awarded a CPPW grant to promote policy changes for obesity prevention, and Lenoir County is the setting for a heart disease prevention program (Heart Healthy Lenoir), in collaboration with the University of North Carolina-Chapel Hill (UNC-CH) Center for Population Health and Health Disparities. The Pitt County CPPW project is funded by a 24-month grant and has 10 objectives for promoting policy and environmental changes related to physical activity and healthy eating. Heart Healthy Lenoir is funded by a 5-year grant and has multiple components, including a lifestyle intervention and policy and environmental changes to support healthy lifestyle choices. Our 2 separate but related policy-assessment strategies evolved from 1 investigator's involvement in both projects.

## The Process: Identifying the Most and Least Winnable Policies

We used 2 processes to identify winnable policies with local stakeholders. In Pitt County in May through September 2011, we conducted 11 face-to-face, individual in-depth interviews with stakeholders, including town and county planners and managers, a school human resources manager, a health promotion dietitian, a mayor, and a city council member. In Lenoir County, in May 2011, we led a group discussion among 19 local stakeholders on a community advisory council, including such leaders as health promotion leaders at the health department and hospital, a county commissioner, and local business people. The council was assembled in August 2010, and members were identified through the local health alliance, chamber of commerce, county school board, and local government. The council had met twice before the meeting we describe.

## Pitt County: Individual In-Depth Interviews

Four of the 11 Pitt County interview participants were members of the CPPW leadership team. Other participants were identified using snowball sampling. We tried to recruit economic leaders in the community (eg, a developer, a surveyor) but did not get responses after multiple contact attempts. We obtained signed informed consent from each participant. Interviews were audio-recorded and transcribed verbatim to facilitate future analysis of key themes related to barriers to and facilitators of policy change, as well as next steps to move health-related policies forward. The project was approved by the East Carolina University Medical Center institutional review board.

Interviews consisted of 4 components: 1) reviewing relevant policy documents, 2) identifying the most and least winnable policy issues using the COCOMO assessment, 3) asking *Community Readiness Handbook* (10) questions to discuss the identified policy issues, and 4) discussing Pitt County's list of emerging issues as related to obesity prevention.

### Reviewing relevant policy documents

To initiate discussion on existing local efforts, we asked participants to identify relevant local policy and planning documents they were currently reviewing or revising. We asked about the most recent versions of such documents and about any updates or revisions projected for the next 18 months (the remaining term of the CPPW project). For example, we asked county and city planners about the planned update of the comprehensive land use plan and about obesity-prevention strategies that might be included.

### Identifying most and least winnable policy issues using the COCOMO assessment

We used the 24 COCOMO recommended strategies to develop an assessment tool to facilitate discussion with each participant to identify the most and least winnable policies (Appendix A). The 24 strategies did not always apply to the participant’s expertise, so we tailored the instrument to each participant’s scope of work and jurisdiction. For example, when interviewing the school human resources manager, we selected COCOMO strategies related to school wellness (eg, “Communities should improve availability of healthier food and beverage choices in public service venues”). First, we asked participants to score each COCOMO recommendation according to how realistic it was given the community’s 1) culture; 2) infrastructure, both physical (eg, land use patterns or availability of resources) and intangible (eg, connections between community groups); 3) extent of leadership support, including political will and priorities; and 4) extent of funding support. The most positive responses were scored the highest (eg, very realistic = 4, a lot of funding = 4), and the most negative responses were scored the lowest (eg, very unrealistic = 1, no funding = 1). We tallied the responses to identify the highest scoring strategy as the most winnable and the lowest scoring strategy as the least winnable policy option.

### Asking *Community Readiness Handbook* questions

We selected a subset of questions in the *Community Readiness Handbook* (10) (Appendix B) to facilitate discussion with participants on the most and least winnable policy options identified. The questions included items on community support, leadership support, and potential funding sources. The *Community Readiness Handbook* includes a rubric for scoring questions, but we did not score the questions; instead, we used them only to facilitate discussion among community leaders and identify additional community leaders to interview. We identified 7 additional interview participants on the basis of recommendations from initial interview respondents.

### Discussing Pitt County's list of emerging issues

A list of emerging issues was formulated by the Pitt County Planning Department and included issues that may influence future land use and planning decisions. Separately from the COCOMO assessment, we asked the planners interviewed to discuss issues that might relate to obesity prevention. Three issues were clearly related to obesity prevention ("Ensuring and promoting interconnectivity between developments," "Ensuring that land use patterns benefit community health by providing access to healthy foods, biking/walking trails," and "Providing safe routes to schools from surrounding developments"); others were not obviously related (eg, "Delineation of agricultural/open/natural resource areas based upon new floodplain maps," "Supporting development of voluntary agriculture districts and agricultural land use plan to preserve and protect prime farmland areas"). Our intent was to examine participants' views on the relationships between the issues and obesity prevention.

## Lenoir County Group Discussion Among Stakeholders on a Community Advisory Council

We used the COCOMO assessment during a community advisory council meeting in Lenoir County as a part of the Heart Healthy Lenoir project. At the beginning of the meeting, we asked council members to complete the COCOMO assessment. We introduced the exercise as we did in Pitt County (Appendix A). After all council members had completed the assessment, we scored the recommendations, shared our findings with council members, and asked them to identify facilitators and barriers to the least winnable strategy and the most winnable strategy. We also asked them to identify additional stakeholders to engage in discussion. We did not audio-record the community advisory council discussion, but a research assistant took detailed notes. The Lenoir County COCOMO assessment was reviewed and approved by the UNC-CH institutional review board.

## Refining the COCOMO Assessment Process

We learned lessons from both processes and made changes to the COCOMO assessment process. For example, the first Pitt County community stakeholder interviewed noted that the lowest-scoring recommendations on the COCOMO assessment were worth discussion. We used both the highest and lowest scoring recommendations to facilitate discussion in subsequent interviews. One Pitt County stakeholder asked us to define "underserved" communities, which we defined as low-income and rural areas with less access to healthy foods and physical activity opportunities than other areas. A Lenoir County stakeholder asked us to define "infrastructure," which we defined as roads and physical environmental structures. We then asked council members to offer their own definition. They agreed with our definition but added that the term could also refer to "communication between organizations."

## Potential Adaptations to COCOMO to Improve Application in Rural Areas

COCOMO may need to be adapted for use in rural areas. Many of the recommended measures in our study focused on census tracts. Rural areas may have large census tracts because of low population density; therefore, potential disparities may be "diluted" or "masked." Focusing on 1 large census tract may also omit details on disparities in resource allocation in rural municipalities. For example, a park in a large, rural census tract may be near a higher income development and far from lower-income areas; when calculating metrics such as parks per census tract, this disparity would be masked by the large land area and low population density of the rural tract. Also, many recommendations focus on 1 school district, which also may mask disparities. For example, although a County's school district may adopt healthy school food and beverage policies, adoption and implementation of such policies may be unequal in urban areas that may have more substantial tax base and resources than do rural areas of the same county. In addition, the measures suggested for public transit do not typically apply in rural areas, which have extensive land mass with sparse population density and are not often able to sustain sufficient ridership rates.

COCOMO guidance documents could be improved by providing concrete examples or more specific definitions of "local jurisdiction," because 1 county may include several jurisdictions (towns, cities, and the county), and by providing examples of policies in "public service venues," which are often referenced in the recommended strategies. Finally, COCOMO does not include benchmarks or guidelines for "high" versus "low" values for "supermarkets per 10,000 residents" and "annual farmer's market days per 10,000 residents." Such benchmarks would be useful for future policy development and should incorporate recommendations on the customer base needed to sustain markets, which may vary for urban versus rural municipalities. Although we do not offer recommendations for specific measures, our experience and ideas can help others frame COCOMO adaptations for rural areas.

## Home Rule and Dillon's Rule: Implications for Policy Change

We learned about the distinction between "Home Rule" and "Dillon's Rule" states, which may make obesity prevention policies more or less difficult to implement in local municipalities. When municipalities are granted Home Rule by states, they are allowed more authority to change policies. In Dillon's Rule states, local municipalities may exercise only certain powers expressly delegated by state law (11). Diller and Graff (11) offer suggestions for navigating municipal authority to plan and enact obesity prevention policies for food retail, noting the difficulty of enacting such policies in a Dillon's Rule state. Owens (12) notes the evolution of state legislature over the past century, granting local municipalities increased authority to enact smart-growth policies, despite Home Rule and Dillon's Rule. Although North Carolina is a Dillon's Rule state, (13) the Pitt County planners we interviewed did not report that Dillon's Rule affected their ability to decide and act on obesity prevention policy.

## Next Steps and Conclusions

In rural areas, health-promoting policies and economic development goals may be perceived as being at odds, making such policies challenging to implement and adopt. Common perceptions may be that "rural sprawl" (low-density residential development or commercial strip development in rural areas [14]) results from a healthy economy (15). However, sprawl is associated with negative health outcomes (16,17). Policy change is an intense process, and stakeholders need a simple process by which they can agree on policy changes that have the greatest likelihood of success in a rural community.

Using COCOMO to develop a community-driven approach to identify winnable local policy initiatives for obesity prevention enabled us to learn more about the applicability of COCOMO in 2 eastern North Carolina counties. Our approach may help others to lay foundations for public health professionals, researchers, and local stakeholders to partner and identify, adopt, and implement effective and feasible obesity prevention policies.

## Figures and Tables

**Table.** Characteristics of Residents of Pitt and Lenoir Counties vs the State of North Carolina, 2006

**Characteristic**	**Pitt County**	**Lenoir County**	**North Carolina**
Per capita income, $^a^	21,017	20,965	32,234
% Overweight^a^	36	41	37
% Obese^a^	30	33	25
5-Year diabetes mortality rate, per 100,000^b^	35.09	40.17	25.91
5-Year heart disease mortality rate, per 100,000^b^	213.57	277.18	296.54

a Sources: Pitt County Health Department (6) and Huff (7).

b Source: East Carolina University (8). Rates are from 2003 through 2007.

## Appendices

### Appendix A. Modified COCOMO Assessment

The following instructions were provided to Pitt County in-depth interview participants: "The purpose of this activity is to identify potential "winnable" obesity prevention policies in Pitt County, using a set of measures developed by the Centers for Disease Control and Prevention called COCOMO, which stands for the Common Community Measures for Obesity Prevention. Please circle the response under each COCOMO Recommendation based on your opinion of how realistic it is for the culture and infrastructure of Pitt County, the extent of leadership, and funding support. When you see the word 'funding,' think broadly about not just city or county funding, but about other sources of funding (grants, private foundations, etc). When you see the word 'underserved,' we are talking about low-income and rural areas, which might not have as much access to healthy foods and physical activity opportunities as other areas." Each recommendation was assigned a "Winnability Score" on the basis of respondents" answers.

**Table:**

**COCOMO Recommendation/Interview Question**	Response Option
**1. Communities should increase availability of healthier food and beverage choices in public service venues.**	
How realistic given the community culture?	Very realistic/Somewhat realistic/Somewhat unrealistic/Very unrealistic
How realistic given the community infrastructure?	Very realistic/Somewhat realistic/Somewhat unrealistic/Very unrealistic
To what extent do community leaders support this recommendation?	A lot of support/Some support/Very little support/No support
To what extent is there current funding for this recommendation?	A lot of funding/Some funding/Very little funding/No funding
**2. Communities should improve availability of affordable healthier food and beverage choices in public service venues.**	
How realistic given the community culture?	Very realistic/Somewhat realistic/Somewhat unrealistic/Very unrealistic
How realistic given the community infrastructure?	Very realistic/Somewhat realistic/Somewhat unrealistic/Very unrealistic
To what extent do community leaders support this recommendation?	A lot of support/Some support/Very little support/No support
To what extent is there current funding for this recommendation?	A lot of funding/Some funding/Very little funding/No funding
**3. Communities should improve geographic availability of supermarkets in underserved areas.**	
How realistic given the community culture?	Very realistic/Somewhat realistic/Somewhat unrealistic/Very unrealistic
How realistic given the community infrastructure?	Very realistic/Somewhat realistic/Somewhat unrealistic/Very unrealistic
To what extent do community leaders support this recommendation?	A lot of support/Some support/Very little support/No support
To what extent is there current funding for this recommendation?	A lot of funding/Some funding/Very little funding/No funding

### Appendix B. *Community Readiness Handbook* Interview Questions (10)

**Table:**

**Community Efforts and Community Knowledge of Efforts**
Using a scale from 1 to 10, how much of a concern is this issue in your community (with 1 being "not at all" and 10 being "a very great concern")? Please explain.
Please describe the efforts that are available in your community to address this issue.
How long have these efforts been going on in your community?
What does the community know about these efforts or activities?
What are the strengths of these efforts?
What are the weaknesses of these efforts?
What formal or informal policies, practices, and laws related to this issue are in place in your community, and for how long? [Prompt: An example of "formal" would be established policies of schools, police, or courts. An example of "informal" would be similar to the police not responding to calls from a particular part of town.]
Are there segments of the community for which these policies, practices, and laws may not apply? [Prompt: For example, due to socioeconomic status, ethnicity, age, etc.]
Is there a need to expand these policies, practice,s and laws? If so, are there plans to expand them? Please explain.
Is there a need to expand these policies, practices, and laws? If so, are there plans to expand them? Please explain.
How does the community view these policies, practices, and laws?
**Leadership**
Who are the "leaders" specific to this issue in your community? [Who else should we speak to about this issue?]
Using a scale from 1 to 10, how much of a concern is this issue to the leadership in your community (with 1 being "not at all" and 10 being "of great concern")? Please explain.
How are these leaders involved in efforts regarding this issue? Please explain. (For example, are they involved in a committee, task force, etc? How often do they meet?)
Would the leadership support additional efforts? Please explain.
**Community Climate**
How does the community support the efforts to address this issue?
What are the primary obstacles to efforts addressing this issue in your community?
Based on the answers that you have provided so far, what do you think is the overall feeling among community members regarding this issue?
**Knowledge About the Issue**
How knowledgeable are community members about this issue? Please explain. (Prompt: For example, dynamics, signs, symptoms, local statistics, effects on family and friends, etc.)
What type of information is available in your community regarding this issue?
What local data are available on this issue in your community?
How do people obtain this information in your community?
